# Role of Advanced Glycation End Products in Mediating Glycated Haemoglobin and Pulse Wave Velocity in Healthy Adults

**DOI:** 10.3390/biomedicines13010137

**Published:** 2025-01-08

**Authors:** Irene Martínez-García, Alicia Saz-Lara, Carlos Pascual-Morena, Ana Díez-Fernández, Sara Valladolid-Ayllón, Bruno Bizzozero-Peroni, Óscar Martínez-Cifuentes, Eva Rodríguez-Gutiérrez, Iván Cavero-Redondo

**Affiliations:** 1 CarVasCare Research Group, Facultad de Enfermería de Cuenca, Universidad de Castilla-La Mancha, 16071 Cuenca, Spain; irene.mgarcia@uclm.es (I.M.-G.); saravalladolid0@gmail.com (S.V.-A.); osmaci@hotmail.com (Ó.M.-C.); ivan.cavero@uclm.es (I.C.-R.); 2 Health and Social Research Center, Universidad de Castilla-La Mancha, 16071 Cuenca, Spain; carlos.pascual@uclm.es (C.P.-M.); ana.diez@uclm.es (A.D.-F.); bruno.bizzozero@uclm.es (B.B.-P.); eva.rodriguez@uclm.es (E.R.-G.); 3 Facultad de Enfermería de Albacete, Universidad de Castilla-La Mancha, 02006 Albacete, Spain; 4 Hospital General Universitario de Elda, 03600 Elda, Spain; 5 Higher Institute of Physical Education, Universidad de la República, Rivera 40000, Uruguay; 6 Hospital Universitario Mutua Terrassa, 08221 Terrassa, Spain; 7 Research Network on Chronicity, Primary Care and Health Promotion (RICAPPS), 16071 Cuenca, Spain

**Keywords:** advanced glycation end products, skin autofluorescence, glycated haemoglobin, pulse wave velocity, arterial stiffness, healthy adults, mediation, cross-sectional

## Abstract

**Background/Objectives**: Poor metabolic control is associated with increased levels of advanced glycation end products (AGEs), which in turn may lead to increased arterial stiffness. The aim of this study was to estimate the association between glycated haemoglobin A1c (HbA1c) and aortic pulse wave velocity (a-PWV) in healthy subjects and to analyse the mediating effect of AGEs measured by skin autofluorescence (SAF) on this association. **Methods**: HbA1c, a-PWV and SAF were analysed in 390 healthy Spanish subjects from the EVasCu study (42.02 ± 13.14 years, 63.08% females). A directed acyclic graph (DAG) was generated to define the covariates to be included, and the model was confirmed via multiple linear regression analysis. Descriptive and exploratory analyses were performed to investigate the associations between variables. Finally, adjusted and unadjusted mediation analyses were performed to verify the influence of SAF on the main association between HbA1c and a-PWV. **Results**: Multiple linear regression analyses for a-PWV supported the validity of the structure in the DAG. Descriptive and exploratory analyses revealed that when the models were adjusted to include all covariates, the statistical significance of the main association disappeared. Mediation analysis revealed that SAF mediated 35.77% of the effect of HbA1c on a-PWV in the unadjusted model and 42.18% after adjusting for covariates. **Conclusions**: Our study suggests that increases in HbA1c levels are associated with increases in a-PWV and that this relationship is mediated by the SAF score in healthy adults.

## 1. Introduction

The prevalence of cardiovascular disease (CVD) has doubled worldwide in the last 20 years, leading to an increased number of deaths and a significant increase in years of life lost, disability-adjusted life years, and years lived with disability [1]. Arterial stiffness is a key biomarker of cardiovascular pathophysiology [2,3] and is influenced by several factors, such as aging and sex [4,5]. In addition, it is affected by impaired glucose metabolism, with persistent hyperglycaemia being one of the causative mechanisms of arterial stiffness, contributing to the subsequent increase in cardiovascular risk (CV risk) [6,7].

Currently, a multitude of validated non-invasive methods are available to measure arterial stiffness, which provide useful biomarkers in the clinical setting [8,9]. Among these, pulse wave velocity (PWV) is regarded as the gold standard for measuring arterial stiffness [9]. In clinical practice, oscillometric assessment of PWV is advocated because of its simplicity, reduced variability and independence from specific distance measurements [10]. In addition, the aortic pulse wave velocity (a-PWV) has been proposed for CV risk assessment because of its high predictive ability for CVD risk [11]. On the other hand, the gold standard for assessing glycaemic control is glycated haemoglobin A1c (HbA1c) [12], which is considered a better glycaemic indicator than fasting plasma glucose [13]. In 2010, the American Diabetes Association proposed it as a diagnostic criterion for diabetes and has maintained it to date, citing its accuracy and stability [14,15].

Several studies have reported a significant association between PWV and HbA1c across different demographic groups, including the Spanish population [13,16,17]. However, important gaps in the understanding of this association remain, as the underlying biological mechanisms are not fully understood. Several pathophysiological pathways associated with HbA1c, such as arterial wall thickness, advanced protein glycation, stress and/or chronic inflammation, influence arterial stiffness [6,18,19]. In particular, advanced protein glycation and the subsequent accumulation of advanced glycation end products (AGEs) may play key mediating roles in this relationship [6], although this interpretation is speculative and needs to be confirmed. AGEs’ accumulation has been associated with increased vascular damage [20], in addition to increased AGEs’ accumulation in individuals with diabetes as a result of chronic hyperglycaemia and oxidative stress [21,22]. In the pathogenesis of arterial stiffness, protein glycation and AGEs’ formation are intermediate steps that may explain the CV risk associated with previous increases in HbA1c in individuals with diabetes [23]. These findings suggest that AGEs’ accumulation could be an underlying mechanism determining the relationship between HbA1c and PWV. Skin autofluorescence (SAF) is a non-invasive, rapid and simple technique for the direct measurement of AGEs’ accumulation that has been widely validated and reproduced in different populations, including healthy individuals, patients with diabetes and different ethnic groups [20,24,25]. Despite its proven accuracy and efficacy, it is important to consider specific factors that may influence the test results, particularly age, which has been shown to significantly affect SAF measurements [24].

To our knowledge, there are no reports exploring the possible mediating role of SAF in the relationship between HbA1c and a-PWV. Although previous studies have speculated on this possibility [6], research has been limited to exploring bivariate relationships between parameters without considering all three simultaneously [6,17,20,21]. Mediation analysis provides information on the association between two factors, in this case, HbA1c and a-PWV, and the extent to which a third variable, SAF, mediates the main association. Furthermore, the study of this relationship in subjects without diagnosed pathologies could be highly important since it could identify direct and intermediate or mediating factors at an early stage, contributing to improving the design of preventive strategies for cardiovascular health. Because of the need to rationally substantiate the mechanism of arterial stiffness, this study was conducted with the objective of assessing the associations among the variables HbA1c, a-PWV and SAF, as well as the ability of the SAF to mediate the main association between HbA1c and a-PWV in healthy adults.

## 2. Materials and Methods

### 2.1. Study Design

This study is based on data from the cross-sectional EVasCu study in Spain, which was designed to validate a model of early vascular ageing (EVA) as an index of CV risk in healthy adults via confirmatory factor analysis [26]. This study was approved by the Clinical Research Ethics Committee of the Cuenca Health Area (REG: 2022/PI2022) and was conducted according to the standards of the Declaration of Helsinki. This research was conducted following the Guideline for Reporting Mediation Analyses (Supplementary Table S1) and the guidelines for reporting observational studies “Strengthening the Reporting of Observational Studies in Epidemiology (STROBE) Statement” (Supplementary Table S2) [27,28].

### 2.2. Participants

The sample size was calculated via Epidat software 4.2, which yielded a sample size of 355 healthy participants, an estimated effect of 1, an alpha risk of 0.05 and an absolute precision level of 0.04 to detect a statistically significant result for the Early Vascular Aging Index (EVA Index) [29]. The EVA Index is used to assess vascular health and identify signs of accelerated arterial aging, making it clinically relevant in the early detection of cardiovascular risk factors. The effect size of 1 was determined on the basis of previous studies to ensure that the calculation had sufficient statistical power to allow the study to detect significant differences between individuals. Finally, 390 participants were enrolled, a number that exceeded the sample size originally calculated to allow for potential dropouts or study variability.

The participants volunteered for the study after the project was advertised through various media and social networks. The inclusion criteria were as follows: over 18 years of age, clinically stable for the previous 6 weeks and provided written consent to participate in the study. Subjects enrolled in other trials of any intervention, those receiving pharmacological treatment (such as hypoglycaemic drugs, lipid-lowering drugs, antihypertensive drugs, etc.) or those with a previous diagnosis of CVD (such as diabetes, arterial hypertension, myocardial infarction or stroke) were excluded from the study. The recruitment and measurement period lasted from June 2022 to December 2022 at the Health and Social Research Centre of the University of Castilla-La Mancha.

### 2.3. Dependent Variable

Arterial stiffness was assessed by a-PWV via the non-invasive oscillometric technique Mobil-O-Graph^®^ (IEM GmbH, Stolberg, Germany). This method has been shown to provide values with an acceptable level of accuracy compared with intra-aortic measurements [30]. The validated ARCSolver algorithm (Austrian Institute of Technology GmbH, Vienna, Austria) was used [31,32]. Measurements were performed in a quiet environment after a five-minute rest period, during which the participant was asked to avoid caffeine, smoking or physical activity for at least 30 min prior to measurement. With the participant seated with their legs uncrossed and feet flat on the floor, a cuff appropriate for the size of the non-dominant arm was placed approximately 2 cm above the elbow. The arterial symbol was placed over the brachial artery, and the pressure tube was passed unbent behind the neck. After entering the patient’s age, sex, weight, height and smoking habits into the software, the participant was instructed not to speak during the measurement. The device inflates the cuff and records the oscillations of the brachial pulse wave, providing pulse wave analysis, along with measurements of brachial blood pressure parameters, central blood pressure, augmentation pressure, the augmentation index, peripheral vascular resistance and cardiac output.

### 2.4. Independent Variable

Venous blood samples were taken from participants between 8:00 and 9:00 a.m. The participants were instructed to fast overnight, with no food or caloric drinks for 12 h prior to blood extraction. Venipuncture was performed via the antecubital vein of each participant. Blood samples were collected and stored according to established protocols and then sent to the laboratory of the Virgen de La Luz Hospital in Cuenca for subsequent analysis. The samples were handled in a controlled manner to maintain their integrity and obtain accurate laboratory results. HbA1c was determined by high-performance liquid chromatography using the ADAMS A1c analyser HA-8180 V (ARKRAY, Inc., Kyoto, Japan).

### 2.5. Mediating Variable

AGEs were measured by SAF using the AGE Reader (DiagnOptics TechnologiesBV, Groningen, The Netherlands). As most AGEs are autofluorescent, this device performs an estimation of AGE levels via the SAF [33]. The device emits an excitation light of 300–420 nm, which is directed towards the skin of the forearm. This light excites the fluorescent fractions of the tissue, which then emit a different wavelength. The emitted light is detected by a spectrometer using a specific range where the greatest contribution of fluorescence is derived from AGEs that are bound to collagen, in addition to AGEs that are bound to other proteins and lipids [33]. The approximate measurement time is 20 s. The device performs three measurements on each arm and calculates the mean by obtaining a value for each arm. The mean of the two measurements was calculated for each subject. Measurements were taken on both arms, avoiding skin imperfections such as scars, moles, tattoos and birthmarks, and the resulting average was taken from both arms. The participants were asked not to use any skin creams prior to the measurement to avoid interference with the results [34]. In cases where creams were used, the skin of the forearm was cleaned with soap and water and dried before measurement. In addition, the light in the room was controlled, and a dim room was chosen for all measurements. The amount of light in the room was controlled to avoid interference from any external light source, both natural and artificial. The SAF values are expressed in arbitrary units.

### 2.6. Potential Confounding Variables: Adjustment of Variables

To identify the minimum sufficient adjustment set (MSAS) for estimating the total effect of HbA1c on a-PWV, a theoretical causal diagram was constructed on the basis of an exhaustive review of the existing scientific literature [3,4,5,6,7,17,18,19,20,21,22,23,24]. A directed acyclic graph (DAG) was subsequently constructed [35] via the online tool DAGitty [36]. The covariates age, sex, smoking status, insulin level, systolic blood pressure (SBP), body mass index (BMI) and socioeconomic status were identified as the MSAS in the model, according to the backdoor criterion [37] (Supplementary Figure S1).

Age, sex and smoking status were collected via self-report questionnaires. For sex, the possible answers were men or women. Smoking status was classified as follows: (i) smoker; (ii) ex-smoker of 0–1 years; (iii) ex-smoker of 1–5 years; (iv) ex-smoker of more than 5 years; and (v) nonsmoker.

Insulin was measured in serum blood samples, which were collected via cubital vein puncture between 8:15 and 9:00 a.m. after at least 12 h of fasting. Blood samples were collected and stored according to established protocols and then sent to the laboratory of the Virgen de La Luz Hospital in Cuenca for subsequent analysis. The samples were processed on the Roche Diagnostics COBAS C711 system (Roche Diagnostics International Ltd., Rotkreuz, Switzerland), the blood glucose concentration was determined via the hexokinase method, and the fasting insulin concentration was determined via a one-step chemiluminescent microparticle immunoassay via a processing platform consisting of two Abbott Laboratories ARCHITECTi2000SR systems (Abbott Park, IL, USA).

The participants’ height and weight were measured twice via a SECA 222 hand-held measuring rod (Seca^®^ GmbH & Co. KG, Hamburg, Germany) and a SECA 869 scale (Seca^®^ GmbH & Co. KG, Hamburg, Germany). The mean of the two measurements was used for analysis. BMI was calculated as kg/m^2^.

SBP was measured in a quiet place and after a 5 min rest period using the Omron^®^ M5-I monitor (Omron Healthcare UK Ltd., Buckinghamshire, UK), with a cuff size corresponding to the participant’s arm circumference. SBP was measured twice, five minutes apart, and the mean of the two measurements was used for analysis.

Educational level was measured via a self-report questionnaire with 6 response options: (i) unable to read or write; (ii) incomplete primary education; (iii) primary education; (iv) secondary education; (v) professional education/higher education; and (vi) university education. Educational level has been recognised as a socioeconomic determinant of CV risk [38] and was therefore included as a covariate.

In addition, the assessment of CV risk derived from the SAF was considered. The CV risk derived from the SAF classifies risk into 4 levels: no increased CV risk; risk group I: limited increase in CV risk; risk group II: increased CV risk; and risk group III: definite CV risk.

### 2.7. Statistical Methods

Both statistical (Kolmogorov–Smirnov test) and graphical (normal probability plots) methods were used to examine the fit to a normal distribution for each continuous variable. Sex differences in descriptive data were assessed via Student’s *t*-test for continuous descriptive variables and the chi-square test for categorical descriptive variables. The homogeneity of variances was assessed via Levene’s test, and scatterplots of the residuals and multicollinearity were generated via variance inflation factors (VIFs).

To evaluate and validate the structure proposed by the DAG, two multiple regression analyses of a-PWV were performed for the unadjusted model and the model adjusted by the MSAS. In addition, local weighted dispersion smoothing (LOESS) regression was performed to assess the association between HbA1c and SAF, considering a CV risk variable provided by the SAF. According to the form of LOESS regression, a subsample excluding subjects classified as risk group III (definite CV risk) was created and identified as potential outliers.

Partial and bivariate Pearson correlation coefficients (MSAS adjusted and unadjusted) were calculated to examine the relationships between HbA1c, a-PWV and SAF. These calculations were performed for the full sample as well as for the subsample without definite CV risk to explore possible differences in the relationships between variables on the basis of participant characteristics.

To explore the variation in a-PWV according to HbA1c exposure levels, HbA1c was categorised into tertiles. Analyses of variance and covariance were subsequently performed for HbA1c tertiles (Model 0), controlling for SAF (Model 1), controlling for MSAS (Model 2) and controlling for MSAS and SAF (Model 3). These analyses were performed both in the full sample and in the subsample by excluding participants identified as having definite CV risk according to SAF risk.

Therefore, mediation analyses were performed via the PROCESS macro for SPSS, version 3.5 [39], with Model 4 selected to assess the possible mediation of SAF in the association between HbA1c and a-PWV, both in the full sample and in the subsample excluding participants with definite CV risk. In addition, to explore possible sex differences, mediation analyses were conducted separately for men and women. Mediation analyses were performed both with the unadjusted model to assess the direct and indirect relationships between the three variables of interest and with the adjusted MSAS model to ensure more accurate and robust estimates. The purpose was to examine the total effect (c) and the direct effects (a, b, c’) to determine the unstandardised regression coefficient and the significance between the independent and dependent variables of the selected model. Similarly, the indirect effect (EI) obtained from the product of the coefficients (a × b) was calculated, showing the change in the a-PWV variable for each unit change in HbA1c that is mediated by the SAF. To test the mediation hypothesis, this macro uses bootstrapping methods as recommended by Preacher and Hayes [40] via a 5000-sample bootstrap resampling procedure. Estimates and confidence intervals (95%) were calculated for the EI, considering the point estimate as the mean when the confidence interval did not contain zero. Finally, the percentage of mediation was calculated by dividing the indirect effect (a × b) by the total effect (c). Following Hayes’ recommendation, the concepts of complete mediation and partial mediation were not used in this study [39]. The missing data were not considered in the analyses.

A two-sided statistical significance criterion of *p* ≤ 0.05 was used. IBM SPSS 28 software (IBM Corp., Armonk, NY, USA) was used for all the statistical analyses. STATA 15.1 software was used to generate the LOESS scatterplot.

## 3. Results

The study flowchart is shown in detail in Supplementary Figure S2. A total of 390 healthy adults were included, with a mean age of 42.02 ± 13.14 years, and 63.08% were female. The characteristics of the participants are described in Table 1, which shows the results for the full sample and is stratified by sex.

The results of the multiple linear regression analyses for the a-PWV supported the validity of the structure proposed in the DAG (Supplementary Table S3). Significance was observed for all variables except for smoking status, educational level and HbA1c (in the MSAS-adjusted model). All the VIFs were lower than 2, so it was concluded that there was no collinearity between the variables. Both models showed adequate summary statistics (significant ΔF, adequate R2 and Durbin–Watson close to 2), but an improvement in all statistics was observed in the covariate-adjusted model, which improved the model (higher ΔF) and decreased the autocorrelation of the residuals (Durbin–Watson close to 2), with 90% of the variability of the a-PWV in the covariate-adjusted model being explained by the covariates, compared with 30% in the unadjusted model.

**Figure 1:**
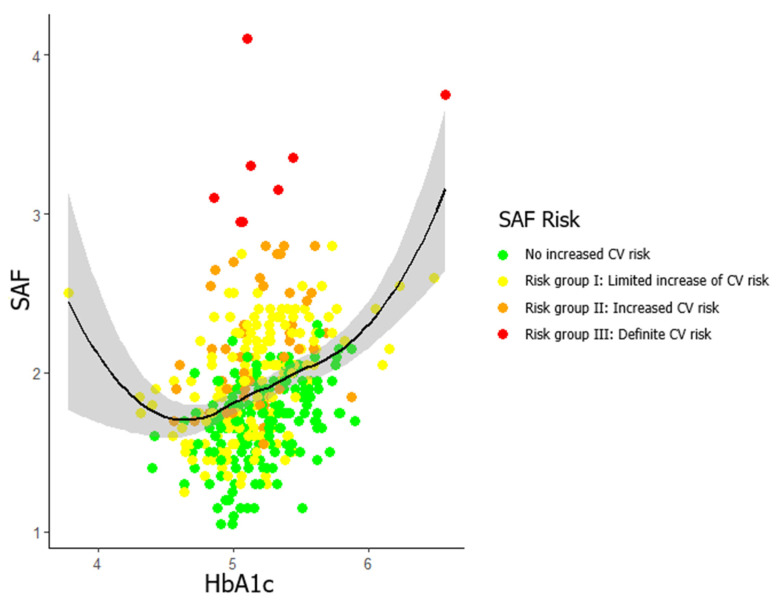
Scatterplot illustrating LOESS regression analysis between skin autofluorescence and HbA1c, including SAF cardiovascular risk. Observations: CV risk, cardiovascular risk; HbA1c, glycated haemoglobin; SAF, skin autofluorescence.

Figure 1 shows the association between HbA1c and SAF, considering the CV risk provided by SAF, and reveals that individuals in risk group III had atypical patterns. Consequently, a subsample excluding these individuals was used, and further analyses were performed on this subsample.

Table 2 shows the bivariate and partial correlation coefficients (r) between HbA1c, SAF and a-PWV for the full sample and the subsample. In unadjusted Model 1, significant associations were found between all model variables (*p* < 0.05). However, in Model 2 adjusted by the MSAS, a loss of significance was found in the main association between HbA1c and a-PWV.

Table 3 shows the mean differences in the overall a-PWV score by HbA1c tertiles in both the full sample and the subsample, with significant differences in Model 0 (raw data analysis) and Model 1 (controlling for SAF) but disappearing in Model 2 (controlling for MSAS) in Model 3 (controlling for MSAS and SAF).

**Figure 2:**
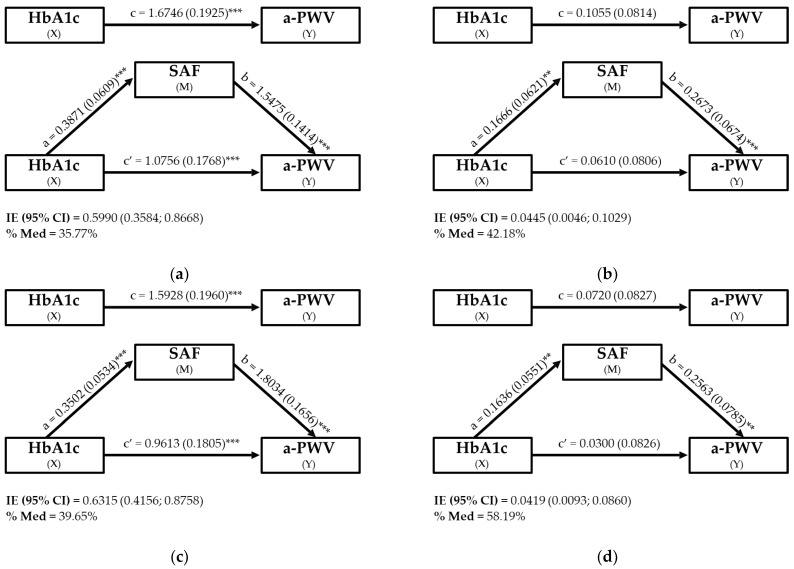
Mediation effects of skin autofluorescence (SAF) on the relationship between HbA1c and a-PWV across four analyses: (**a**) full sample unadjusted, (**b**) subsample unadjusted, (**c**) full sample adjusted for covariates, (**d**) subsample adjusted for covariates. Observations: The variables in the mediation model were HbA1c (X), SAF (M) and a-PWV (Y). The adjusted model is adjusted by the minimum sufficient adjustment set provided by the directed acyclic graph, which was corroborated with the multiple regression model for a-PWV. The subsample was derived from the full sample by excluding participants identified as having definite CV risk according to SAF risk. The results showed unstandardised beta coefficients and error standards. Statistical significance is indicated by asterisks, where ** indicates *p* ≤ 0.01 and *** indicates *p* ≤ 0.001. Abbreviations: % Med: mediation percentage; a-PWV, aortic pulse wave velocity; HbA1c, glycated haemoglobin; IE (95% CI): indirect effect (95% confidence interval); IM: mediator variable; SAF, skin autofluorescence; X, independent variable; Y, dependent variable.

On the basis of the above results, mediation analyses were carried out on both the full sample and the subsample, unadjusted and adjusted for MSAS (Figure 2). For the two unadjusted models (Figure 2a,b), significance was found in all the model equations (*p* < 0.001). However, in the adjusted models, we found a weakening of significance in routes a and b compared with the unadjusted models (*p* < 0.05) and a loss of significance in routes c and c’. The percentage of the total mediation effect increased in the adjusted models (full sample: 35.77% to 42.18%; subsample: 39.65% to 58.19%), whereas the indirect effect decreased (full sample: 0.60 to 0.04; subsample: 0.63 to 0.04) but maintained significance.

Additional analyses by sex are shown in Supplementary Table S4. In the unadjusted models, a higher percentage of mediation was observed in men than in women. In the covariate-adjusted models, the significant indirect effect was lost in women, and no evidence of mediation was found in this group. In contrast, in men, although the percentage of mediation decreased, mediation remained significant.

## 4. Discussion

To our knowledge, this is the first study to estimate the influence of SAF on the relationship between HbA1c and a-PWV via mediation analysis procedures. Our data support that, in healthy subjects, higher levels of HbA1c are associated with higher levels of SAF and a-PWV. The results of the mediation analysis suggest that SAF explains the association between HbA1c and a-PWV, with a mediation percentage of 35.77% in the full sample. When adjusting for covariates (MSAS), steps c and c’ lost statistical significance and increased the percentage of mediation to 42.18% in the full sample. The reduction in the indirect effect in the adjusted model indicates that covariates explain a part of the variability in a-PWV, which was attributed to HbA1c and SAF in the unadjusted model. The increase in the percentage of mediation after adjustment for covariates (MSAS) reinforces the robustness of the model, even in the presence of confounders.

In the subsample excluding participants identified as being at CV risk according to the SAF risk, similar results were found to those of the full sample. When the analyses were stratified by sex, greater mediation was found in men than in women in unadjusted models. However, when covariates were introduced into the model, different results were found in the sample and subsample. Male sex has previously been described as a determinant factor in the development of arterial stiffness [4]; however, the differences found could be due to hormonal, social or behavioural differences not controlled for in the study. Additionally, the differences observed with respect to sex could be a product of statistical noise in smaller subsamples, reducing the statistical power of the analysis. The effect of sex on a-PWV variability was not the largest according to the multiple linear regression (r = 0.115, *p* = 0.013), so its inclusion as a covariate seems to be more appropriate than the analysis stratified by sex. In addition, the covariate-adjusted regression model (including sex in the MSAS) suggested a good model fit, with 90% of the variability in a-PWV being explained by the covariates.

The association between HbA1c and PWV has been described in previous studies [13,16,17], highlighting the importance of metabolic factors in the modulation of arterial stiffness. A previous study reported that the association between HbA1c and PWV was mediated by insulin, with a mediation percentage of 17.9% [17]. Our study extends this knowledge by showing that SAF plays a significant role in the association between HbA1c and a-PWV, even after accounting for possible confounding factors, including insulin. The link between AGEs’ accumulation and arterial stiffness has been previously established, suggesting the possible involvement of AGEs’ accumulation in the pathophysiology of arterial stiffness [41,42].

AGEs’ accumulation induces cross-linking of extracellular matrix proteins, such as collagen and elastin, altering their structure and elasticity and promoting increased oxidative stress and inflammation [43,44]. The resulting cross-links reduce the buffering function of arteries, leading to a subsequent increase in arterial stiffness [45]. The association between AGEs’ accumulation and HbA1c is more controversial, with most studies finding a significant association [46,47,48,49]. The lack of association between HbA1c and AGEs observed could be explained by the shorter turnover time of HbA1c than that of SAF, as well as individual differences in the management of the hyperglycaemic state and associated oxidative stress [49]. It is possible that SAF correlates better with HbA1c from previous years than with the current HbA1c, as SAF represents longer-term cumulative metabolic control [48]. In healthy subjects, the mediating role of SAF in the relationship between HbA1c and a-PWV may be explained by the initial and progressive nature of the pathophysiological processes. In pathological contexts, the relationship becomes more complex, as the accumulation of AGEs represents not only hyperglycaemia but also increased metabolic load, inflammation and oxidative stress [18,50]. A vicious cycle is thus created in which the accumulation of AGEs, in addition to increasing arterial stiffness, creates a scenario conducive to increased AGEs’ formation due to the oxidative stress and inflammation generated [18,51]. Moreover, other additional mechanisms are involved in the pathophysiology of arterial stiffness, generating a multifactorial scenario in which other factors could acquire greater relevance [52].

The results of this study should be interpreted with caution, as some potentially relevant covariates were not included in the analysis, which may affect the generalisability of the findings. Ethnicity and genetic factors may influence SAF [25], but our study consisted of a healthy population of European origin, so the inclusion of this variable was not considered due to the homogeneity of the sample. Previous studies have reported that diet and food preparation may influence some variables, especially AGEs [53,54,55]. In our study, this information was collected via the Mediterranean Diet Adherence Screener questionnaire [56]; however, there could be recall bias, in addition to the questionnaire’s inability to assess in depth the cooking, frequency and quantity of food consumed. The level of physical activity could be another possible confounding factor in the main association [57]. In our study, physical condition was assessed via the International Fitness Scale [58], which is subjective in nature and depends on self-perception when self-reported. Physical activity was also assessed via wearable activity trackers (Xiaomi bands), but the rate of data loss for this variable was significant. These variables were not included in the study as potential confounders, as the DAG plot indicated that they were not included according to the MSAS. In addition, the adjusted model was analysed via multiple linear regression, which revealed that there was no collinearity between the variables and that 90% of the variability in a-PWV was explained by the covariates selected by the DAG (MSAS). This suggests that the selected variables adequately capture the relevant factors for the model.

The results of this study have important clinical and public health implications for the prevention of CVD. The observed association between HbA1c and a-PWV highlights the importance of maintaining optimal glycaemic control in healthy populations as a preventive strategy against the development of atherosclerosis and thus CV risk. In patients with coronary artery disease, the SAF score and a-PWV have previously been proposed as promising markers for risk stratification [59]. Our findings extend this perspective by adding to the evidence suggesting that the SAF may play a key role in the pathophysiology of arterial stiffness and therefore serve as a crucial risk biomarker, even at earlier stages, given its relevance in apparently healthy individuals [41]. This finding is important because the inclusion of SAF as a marker in CV risk prevention and assessment strategies could facilitate the implementation of specific measures to control the AGEs’ accumulation and prevent cardiovascular damage at an early stage before it becomes irreversible. In addition, it could improve primary care in general by facilitating decision making, reducing the burden on the healthcare system and promoting personalised medicine. However, although it is a simple and rapid test, cost–benefit studies are needed to assess its introduction into routine clinical practice.

Some limitations of our study should be recognised. First, the main limitation was the cross-sectional design of the study, as it does not allow for causal inference. Longitudinal and experimental studies are needed to confirm the direction and magnitude of the results and establish the temporal order between the variables. Second, our study included healthy subjects without diagnosed CVDs and of European descent, so the results cannot be extrapolated to other populations. Third, the recruitment method may have influenced the composition of the sample, limiting the representativeness of the study population in relation to the target population. Fourth, some of the participants in our study could have been misclassified as healthy and had previously undiagnosed pathologies. However, to reduce this bias, the analysis was additionally performed on a subsample that excluded individuals at CV risk, and the results were consistent with those of the full sample. Fifth, the limited sample size diminished the ability to clarify the influence of sex on the relationships examined. Sixth, another limitation of our study could be the non-inclusion of confounding variables, such as ethnicity, genetic factors, diet or physical activity, which could bias the model. Finally, in our analysis, SAF was selected as a potential mediator [6]; however, it is necessary to continue studying the role of this variable in the relationship to clarify its possible role, taking into account all the possible confounding variables, especially age and blood pressure, which strongly influence the main model [60].

## 5. Conclusions

Our study revealed that increases in HbA1c levels are associated with increases in a-PWV and that this relationship is mediated by SAF in healthy adults without preexisting disease. These findings are important from a public health perspective, as better glycaemic control may contribute to improved vascular health. Considering SAF as a biomarker of CV risk could help improve prevention and assessment strategies and promote targeted action before the risk is irreversible, as it would allow the identification of cardiovascular damage at an early stage. However, future longitudinal studies are needed to address this issue, allowing causal relationships to be established while accounting for relevant covariates such as age or blood pressure. Furthermore, a larger sample size would facilitate sex-specific analysis, which could elucidate possible differences or improve the understanding of the role of sex in the relationship studied.

## Figures and Tables

**Table 1:** Characteristics of the full sample.

Characteristics	Total (*n* = 390)	Men (*n* = 144)	Women (*n* = 246)	*p*-Value
Age, years				
- Mean Age	42.02 ± 13.14	42.33 ± 12.45	41.84 ± 13.55	0.361
- 18–20 years	13 (3.3)	3 (2.1)	10 (4.1)	
- 20–29 years	77 (19.7)	23 (16.0)	54 (22.0)	
- 30–39 years	56 (14.4)	30 (20.8)	26 (10.6)	
- 40–49 years	116 (29.7)	46 (31.9)	70 (28.5)	
- 50–59 years	100 (25.6)	29 (20.1)	71 (28.9)	
- 60–69 years	25 (6.4)	11 (7.6)	14 (5.7)	
- 70–79 years	3 (0.8)	2 (1.4)	1 (0.4)	0.028 *
Smoking status ^1^				
- Smokers	48 (12.4)	14 (9.8)	34 (13.9)	
- Smokers 0–1 years	7 (1.8)	2 (1.4)	5 (2.0)	
- Smokers 1–5 years	13 (3.4)	5 (3.5)	8 (3.3)	
- Smokers >5 years	73 (18.9)	32 (22.4)	41 (16.8)	
- Non-smokers	246 (63.6)	90 (62.9)	156 (63.9)	0.563
Height, cm	167.78 (±0.09)	176.52 (±0.07)	1.63 (±0.06)	<0.001 ***
Weight, kg	70.26 (±14.57)	80.12 (±12.72)	64.48 (±12.33)	<0.001 ***
BMI, kg/m^2^				
- Mean BMI	24.87 (4.25)	25.68 (±3.55)	24.39 (±4.54)	0.002 **
- Underweight	11 (2.8)	1 (0.7)	10 (4.1)	
- Normal weight	207 (53.1)	65 (45.1)	142 (57.7)	
- Overweight	122 (31.3)	60 (41.7)	62 (25.2)	
- Obesity	50 (12.8)	18 (12.5)	32 (13.0)	0.003 **
BP, mmHg ^1^				
- SBP (±SD)	116.67 (±15.21)	125.09 (±12.77)	111.77 (±14.37)	<0.001 ***
- DBP (±SD)	70.36 (±10.57)	72.27 (±10.33)	69.24 (±10.58)	0.003 **
a-PWV, m/s	6.34 (±1.35)	6.53 (±1.34)	6.24 (±1.35)	0.019 *
Insulin, mUI/L ^1^	8.52 (±6.08)	8.29 (±5.44)	8.65 (±6.43)	0.284
HbA1c, %1				
- Mean HbA1c	5.18 (±0.33)	5.16 (±0.37)	5.19 (±0.31)	0.188
- Low tertile	4.85 (±0.19)	4.80 (±0.24)	4.88 (±0.15)	0.012 *
- Medium tertile	5.17 (±0.07)	5.16 (±0.07)	5.17 (±0.07)	0.229
- High tertile	5.53 (±0.22)	5.55 (±0.28)	5.53 (±0.18)	0.292
Skin autofluorescence, AU ^1^				
- Mean AGEs	1.89 (±0.41)	1.91 (±0.46)	1.88 (±0.38)	0.245
- Risk CV 0: No increase	172 (44.6)	62 (43.4)	110 (45.3)	
- Risk CV 1: Limited increase	153 (39.6)	61 (42.7)	92 (37.9)	
- Risk CV 2: Increased	53 (13.7)	17 (11.9)	36 (14.8)	
- Risk CV 3: Very high	8 (2.1)	3 (2.1)	5 (2.1)	0.763
Educational level ^1^				
- Unable to read or write	-	-	-	
- Incomplete primary education	-	-	-	
- Primary education	4 (1.0)	1 (0.7)	3 (1.2)	
- Secondary education	45 (11.7)	19 (13.3)	26 (10.7)	
- Professional/Higher education	116 (30.1)	40 (28.0)	76 (31.3)	
- University education	221 (57.3)	83 (58.0)	138 (56.8)	0.773

Observations: Values are the means ± standard deviations, except for age categories, smoking status, BMI categories, risk CV and education level, which are shown as absolute numbers and percentages (%). The *p*-values were calculated via Pearson’s chi-square test for categorical variables and the independent samples *t* test for continuous variables, with groups defined by sex (men and women). Statistical significance is indicated by asterisks, where * indicates *p* ≤ 0.05, ** indicates *p* ≤ 0.01 and *** indicates *p* ≤ 0.001. A “^1^” was used to denote variables with missing data for some participants (4 participants lacked information on smoking status, 1 on PA, 7 on insulin levels, 3 on HbA1c, 4 on SAF and 4 on educational level). Abbreviations: a-PWV, aortic pulse wave velocity; AU, arbitrary units; cm: centimetres; BP, blood pressure; CV, cardiovascular; DBP, diastolic blood pressure; HbA1c, glycated haemoglobin; kg, kilograms; m/s: metres per second; mUI/L, milliunits per litre; *p*-value, significance; SD, standard deviation; SBP, systolic blood pressure.

**Table 2:** Bivariate and partial correlation coefficients (r) between HbA1c, SAF and a-PWV for the full sample and the subsample.

Variables		Model 1			Model 2		
		Sample Size	Bivariate Correlation Coefficients		Sample Size	Partial Correlation Coefficients	
		N/n	Full Sample	Subsample	N/n	Full Sample	Subsample
HbA1c and a-PWV	Total Women Men	387/375 245/237 142/138	0.402 *** 0.446 *** 0.363 ***	0.388 *** 0.452 *** 0.315 ***	372/365 236/232 136/133	0.068 0.107 0.001	0.046 0.115 −0.079
HbA1c and SAF	Total Women Men	383/375 242/237 141/138	0.310 *** 0.262 *** 0.373 ***	0.321 *** 0.319 *** 0.332 ***	372/365 236/232 136/133	0.139 ** 0.065 0.236 **	0.156 ** 0.111 0.236 **
SAF and a-PWV	Total Women Men	386/378 243/238 143/140	0.552 *** 0.494 *** 0.640 ***	0.555 *** 0.518 *** 0.619 ***	372/365 236/232 136/133	0.211 *** 1.172 ** 0.285 ***	0.176 *** 0.124 0.277 **

Observations: The values in bold indicate statistical significance; Model 1: bivariate correlations (r) unadjusted; Model 2: partial correlation (r) adjusted for the minimum sufficient adjustment set provided by the directed acyclic graph, which was corroborated with the multiple regression model for a-PWV (excluding sex in the data provided for men and women); statistical significance is indicated by asterisks, where ** indicates *p* ≤ 0.01 and *** indicates *p* ≤ 0.001; the subsample was derived from the full sample by excluding participants identified as having definite CV risk according to SAF risk. Abbreviations: a-PWV, aortic pulse wave velocity; HbA1c, glycated haemoglobin; N: sample size for full sample; n: sample size for subsample; SAF, skin autofluorescence.

**Table 3:** Analysis of variance or covariance of a-PWV by HbA1c categories.

Model for a-PWV		Sample Size	Terciles of HbA1c			*p*-Value
		n	Low (L)	Medium (M)	High (H)	
Full sample	M0	129/129/129	5.805 ± 1.07 ^M,H^	6.218 ± 1.37 ^L,H^	7.018 ± 1.31 ^L,M^	<0.001 ***
	M1	129/127/127	5.805 ± 1.07 ^H^	6.226 ± 1.38 ^H^	7.042 ± 1.30 ^L,M^	<0.001 ***
	M2	127/125/124	5.800 ± 1.07	6.235 ± 1.38	7.023 ± 1.31	0.596
	M3	127/123/122	5.800 ± 1.06	6.244 ± 1.39	7.048 ± 1.30	0.666
Subsample	M0	128/123/124	5803 ± 1.08 ^M,H^	6196 ± 1.37 ^L,H^	6989 ± 1.26 ^L,M^	<0.001 ***
	M1	128/123/124	5803 ± 1.08 ^H^	6196 ± 1.37 ^H^	6989 ± 1.26 ^L,M^	<0.001 ***
	M2	126/120/119	5.798 ± 1.07	6.202 ± 1.38	6.993 ± 1.26	0.617
	M3	126/120/119	5.798 ± 1.07	6.202 ± 1.38	6.993 ± 1.26	0.608

Observations: The subsample was derived from the full sample by excluding participants identified as having definite CV risk according to SAF risk. The data are presented as the means ± standard errors (SEs). Statistical significance is indicated by asterisks, where *** indicates *p* ≤ 0.001. Superscript letters indicate statistical significance (*p* < 0.05) between categories for post hoc tests via the Bonferroni comparisons. Abbreviations: a-PWV, aortic pulse wave velocity; HbA1c, glycated haemoglobin, M0: Model 0 (raw data analysis); M1: Model 1 (controlling for SAF); M2: Model 2 (controlling for MSAS); M3: Model 3 (controlling for MSAS and SAF); *p*-value, significance.

## Data Availability

The raw data supporting the conclusions of this article will be made available by the authors, without undue reservation.

## Supplementary Materials

The following supporting information can be downloaded at: https://www.mdpi.com/article/10.3390/biomedicines13010137/s1, Figure S1: Directed acyclic graphs (DAGs) on the causal structure of the relationship between HbA1c (exposure, green) and a-PWV (outcome, blue) and its possible mediating variable, SAF (mediator, blue); Figure S2: Flow chart of the study participants in the current study from the original EVasCu study; Table S1: “Guideline for Reporting Mediation Analyses” checklist for the reporting of mediation analyses of observational studies; Table S2: STROBE Statement. Checklist of items that should be included in reports of cross-sectional studies; Table S3: Results of the multiple linear regression models for the dependent variable (a-PWV), including the full sample: unadjusted model and covariate-adjusted model; Table S4: Mediation effect of SAF on the association between HbA1c and a-PWV, comparing the entire dataset with an additional separate analysis for women and men.
