# Supplementary material for: Role of Advanced Glycation End Products in Mediating Glycated Haemoglobin and Pulse Wave Velocity in Healthy Adults
Source: Biomedicines. 2025 Jan 8;13(1):137. doi: 10.3390/biomedicines13010137 (PMC11759834; doi:10.3390/biomedicines13010137)
Supplement: Supplementary file 1 [file biomedicines-13-00137-s001.zip › biomedicines-3398319-supplementary.pdf]

Role of Advanced Glycation End Products in Mediating Glycated Haemoglobin and Pulse Wave Velocity in Healthy Adults

Irene Martínez-García [1], Alicia Saz-Lara [1,], Carlos Pascual-Morena [2,3], Ana Díez-Fernández [2], Sara Valladolid-Ayllón [1,4], Bruno Bizzozero-Peroni [2,5], Óscar Martínez-Cifuentes [1,6], Eva Rodríguez-Gutiérrez [2,7] and Iván Cavero-Redondo [1]*

Supplementary material

Table S1. "A Guideline for Reporting Mediation Analyses" checklist for the reporting of mediation analyses of observational studies.

Section/Topic	Item Number	Item Description	Reported on page No
**Title and abstract**			
Title	1	Identify that the study uses mediation analysis	1
Abstract	2	Provide a structured summary of the objectives, methods, results, and conclusions specific to mediation analyses	1
**Introduction**			
Background and rationale	3	Describe the study background and theoretical rationale for investigating the mechanisms of interest. Include supporting evidence or theoretical rationale for why the intervention or exposure might have a causal relationship with the proposed mediators. Include supporting evidence or theoretical rationale for why the mediators might have a causal relationship with the outcomes	2
Objectives	4	State the objectives of the study specific to the mechanisms of interest. The objectives should specify whether the study aims to test or estimate the mechanistic effects	2,3
**Methods**			
Study registration	5	If applicable, provide references to any protocols or study registrations specific to the mediation analysis, and highlight any deviations from the planned protocol	NA
Study design and source of data	6	Specify the design of the original study that was used in mediation analyses and where the details can be accessed, supported by a reference. If applicable, describe study design features that are relevant to mediation analyses	3,4
Participants	7	Describe the target population, eligibility criteria specific to mediation analyses, study locations, and study dates (start of participant enrollment and end of follow-up)	3
Sample Size	8	State whether a sample size calculation was conducted for mediation analyses. If so, explain how it was calculated	3
Effects of interest	9	Specify the effects of interest	3-6
Assumed causal model	10	Include a graphic representation of the assumed causal model including the exposure, mediator, outcome, and possible confounders	Figure S1
Causal assumptions	11	Specify assumptions about the causal model	3-6
Measurement	12	Clearly, describe the interventions or exposures, mediators, outcomes, confounders, and moderators that were used in the analyses. Specify how and when they were measured, the measurement properties, and whether blinded assessment was used	3-6

Measurement levels	13	If relevant, describe the levels at which the exposure, mediator, and outcome were measured	5
Statistical methods	14	Describe the statistical methods used to estimate the causal relationships of interest. This description should specify analytical strategies used to reduce confounding, model building procedures, justification for the inclusion or exclusion of possible interaction terms, modelling assumptions, and methods used to handle missing data. Provide a reference to the statistical software and package used	3-6
Sensitivity analyses	15	Describe any sensitivity analyses that were used to explore causal or statistical assumptions and the influence of missing data	6
Ethical approval	16	Name the institutional research board or ethics committee that approved the study. Provide a description of participant informed consent or ethics committee waiver of informed consent	3
**Results**			
Participants	17	Describe baseline characteristics of participants included in mediation analyses. Report the total sample size and number of participants lost during follow-up or with missing data	6,7 Table 1, Figure S2
Outcomes and estimates	18	Report point estimates and uncertainty estimates for the exposure-mediator and mediator-outcome relationships. If inference concerning the causal relationship of interest is considered feasible given the causal assumptions, report the point estimate and uncertainty estimate	7-9, Figure 1, Figure 2, Table 2, Table 3
Sensitivity parameters	19	Report the results from any sensitivity analyses used to assess robustness of the causal or statistical assumptions, and the influence of missing data	10, Table S4
**Discussion**			
Limitations	20	Discuss the limitations of the study including potential sources of bias	12,13
Interpretation	21	Interpret the estimated effects considering the study's magnitude and uncertainty, plausibility of the causal assumptions, limitations, generalizability of the findings, and results from relevant studies	10-12
Implications	22	Discuss the implications of the overall results for clinical practice, policy, and science	12
**Other information**			
Funding and role of sponsor	23	List all sources of funding or sponsorship for the mediation analysis and the role of the funders/sponsors in the conduct of the study, writing of the manuscript, and decision to submit for publication.	13
Conflicts of interest and financial disclosures	24	State any conflicts of interest and financial disclosures for all authors	14
Data and code	25	Authors are encouraged to provide a statement for sharing data and code for the mediation analysis	13

Table S2. STROBE Statement. Checklist of items that should be included in reports of cross-sectional studies.

Checklist strobe	Item	Recommendation	Page
Title and abstract	1	(*a*) Indicate the study's design with a commonly used term in the title or the abstract	1
		(*b*) Provide in the abstract an informative and balanced summary of what was done and what was found	1
**Introduction**			
Background/rationale	2	Explain the scientific background and rationale for the investigation being reported	2
Objectives	3	State specific objectives, including any prespecified hypotheses	2,3
**Methods**			
Study design	4	Present key elements of study design early in the paper	3
Setting	5	Describe the setting, locations, and relevant dates, including periods of recruitment, exposure, follow-up, and data collection	3
Participants	6	(*a*) Give the eligibility criteria, and the sources and methods of selection of participants	3
Variables	7	Clearly, define all outcomes, exposures, predictors, potential confounders, and effect modifiers. Give diagnostic criteria, if applicable	3-5
Data sources/measurement	8	For each variable of interest, give sources of data and details of methods of assessment (measurement). Describe comparability of assessment methods if there is more than one group	3-5
Bias	9	Describe any efforts to address potential sources of bias	3-6
Study size	10	Explain how the study size was arrived at	3
Quantitative variables	11	Explain how quantitative variables were handled in the analyses. If applicable, describe which groupings were chosen and why	3-5
Statistical methods	12	(*a*) Describe all statistical methods, including those used to control for confounding	5,6
		(*b*) Describe any methods used to examine subgroups and interactions	5,6
		(*c*) Explain how missing data were addressed	6
		(*d*) If applicable, describe analytical methods taking account of sampling strategy	5,6

		(*e*) Describe any sensitivity analyses	6
**Results**			
Participants	13	(a) Report numbers of individuals at each stage of study—e.g. numbers potentially eligible, examined for eligibility, confirmed eligible, included in the study, completing follow-up, and analysed	6, Figure S2
		(b) Give reasons for nonparticipation at each stage	6, Figure S2
		(c) Consider use of a flow diagram	6, Figure S2
Descriptive data	14	(a) Give characteristics of study participants (e.g. demographic, clinical, social) and information on exposures and potential confounders	6,7, Table 1
		(b) Indicate number of participants with missing data for each variable of interest	6, Figure S2
Outcome data	15	Report numbers of outcome events or summary measures	6,7, Figure 1 Table 1
Main results	16	(*a*) Give unadjusted estimates and, if applicable, confounder-adjusted estimates and their precision (e.g., 95% confidence interval). Make clear which confounders were adjusted for and why they were included	9,10, Figure 2
		(*b*) Report category boundaries when continuous variables were categorised	Table S3
		(*c*) If relevant, consider translating estimates of relative risk into absolute risk for a meaningful time period	NA
Other analyses	17	Report other analyses done—e.g. analyses of subgroups and interactions, and sensitivity analyses	10, Table S4
**Discussion**			
Key results	18	Summarise key results with reference to study objectives	10,11
Limitations	19	Discuss limitations of the study, taking into account sources of potential bias or imprecision. Discuss both direction and magnitude of any potential bias	12,13
Interpretation	20	Give a cautious overall interpretation of results considering objectives, limitations, multiplicity of analyses, results from similar studies, and other relevant evidence	10-13
Generalisability	21	Discuss the generalisability (external validity) of the study results	12
**Other information**			
Funding	22	Give the source of funding and the role of the funders for the present study and, if applicable, for the original study on which the present article is based	13

Table S3. Results of the multiple linear regression models for the dependent variable (a-PWV), including the full sample: unadjusted model and covariate-adjusted model.

Model for a-PWV (Y)	Independent variables	Descriptive statistics		Regression statistics			Summary statistics for the model			
		n	Mean (SD)/%	r	β (SE)	VIF	R^2	ΔF	p value (ΔF)	DW
Unadjusted	SAF (M)	383	1.889 (0.41)	0.551***	1.547 (0.14)***	1.106	0.366	109.551	<0.001***	1.659
	HbA1c (X)	383	5.181 (0.33)	0.407***	1.076 (0.18)***	1.106				
Adjusted for covariables	SAF (M)	372	1.883 (0.41)	0.560***	0.267 (0.07)***	1.496	0.900	360.709	<0.001***	1.889
	HbA1c (X)	372	5.182 (0.32)	0.421***	0.061 (0.08)	1.309				
	Age	372	(3.2/19.1/14.2/30.4/25.8/6.5/0.8)	0.899***	0.795 (0.02)***	1.681				
	Sex	372	(36.6/63.4)	-0.115*	0.133 (0.05)*	1.274				
	Smoking status	372	(12.4/1.9/3.5/18.8/63.4)	-0.059	0.024 (0.02)	1.054				
	Insulin level	372	8.398 (5.91)	0.135**	0.005 (0.01)	1.438				
	SBP	372	116.71 (15.45)	0.498***	0.026 (0.00)***	1.576				
	BMI	372	24.83 (4.27)	0.368***	0.006 (0.01)	1.621				
	Educational level	372	(0/0/1.1/11.8/29.3/57.8)	-0.084	-0.028 (0.03)	1.094				

Observations: The variable age was categorised into 7 groups by 10-year age ranges, ordered from youngest to oldest: “18-20 years”/”20-30 years”/”30-40 years”/”40-50 years”/”50-60 years”/”60-70 years”/”70-80 years”). The sex variable was categorised as “men”/”women”. The variable smoking status was categorised into “smoker”/“ex-smoker 0–1 year”/“ex-smoker 1–5 years”/“ex-smoker >5 years”/“non-smoker”. The variable educational level was categorised into “cannot read or write”/“no formal education”/“primary education (not completed)”/“completed primary education”/“high school or vocational training”/“university degree”. Statistical significance is indicated by asterisks, where * indicates $p \leq 0.05$, ** indicates $p \leq 0.01$ and *** indicates $p \leq 0.001$.

Abbreviations: ΔF, change in F statistic; β, beta coefficient; a-PWV, aortic pulse wave velocity; BMI, body mass index; DW, Durbin–Watson statistic; HbA1c, glycated haemoglobin; M, mediator variable; n, sample size; p value, significance level; r, Pearson correlation coefficient; R^2, proportion of variance explained by the model in regression analysis; SAF, skin autofluorescence; SBP, systolic blood pressure; SD, standard deviation; SE, standard error; VIF, variance inflation factor; X, independent variable; Y, dependent variable.

Table S4. Mediation effect of SAF on the association between HbA1c and a-PWV, comparing the entire dataset with an additional separate analysis for women and men.

Sample			Total effect X→Y (c path)	Direct effect X + M→ Y (c' path)	X → M (a path)	M→Y (b path)	Indirect effect (a*b)		Effect mediated
**Full sample**		**n**	**Coefficient (SE)**	**Coefficient (SE)**	**Coefficient (SE)**	**Coefficient (SE)**	**Coefficient (SE)**	**95% CI of IE**	**Proportion**
Model unadjusted	Total	383	1.6746 (0.1925)***	1.0756 (0.1768)***	0.3871 (0.0609)***	1.5475 (0.1414)***	0.5990 (0.1300)	0.3584;0.8668	35.77%
	Men	141	1.3662 (0.2882)***	0.5799 (0.2547)*	0.4733 (0.1000)***	1.6615 (0.2006)***	0.7864 (0.2677)	0.3137;1.3655	57.56%
	Women	242	1.9796 (0.2545)***	1.5089 (0.2374)***	0.3236 (0.0769)***	1.4545 (0.1922)***	0.4707 (0.1209)	0.2498;0.7257	23.78%
Model adjusted	Total	372	0.1055 (0.0814)	0.0610 (0.0806)	0.1666 (0.0621)**	0.2673 (0.0674)***	0.0445 (0.0252)	0.0046;0.1029	42.18%
	Men	136	0.0013 (0.1180)	-0.0938 (0.1165)	0.2736 (0.0996)**	0.3477 (0.1005)***	0.0951 (0.0638)	0.0064;0.2488	73.15%
	Women	236	0.1794 (0.1107)	0.1612 (0.1096)	0.0787 (0.0796)	0.2318 (0.0911)*	0.0182 (0.0201)	-0.9196;0.0620	No mediation
**Subsample**		**n**	**Coefficient (SE)**	**Coefficient (SE)**	**Coefficient (SE)**	**Coefficient (SE)**	**Coefficient (SE)**	**95% CI of IE**	**Proportion**
Model unadjusted	Total	375	1.5928 (0.1960)***	0.9613 (0.1805)***	0.3502 (0.0534)***	1.8034 (0.1656)***	0.6315 (0.1180)	0.4156;0.8758	39.65%
	Men	138	1.1665 (0.3010)***	0.4646 (0.2638)	0.3663 (0.0891)***	1.9165 (0.2395)***	0.7019 (0.2470)	0.2508;1.2239	60.17%
	Women	237	1.9735 (0.2539)***	1.3915 (0.2405)***	0.3461 (0.0671)***	1.6816 (0.2217)***	0.5820 (0.1237)	0.3513;0.8380	29.49%
Model adjusted	Total	365	0.0720 (0.0827)	0.0300 (0.0826)	0.1636 (0.0551)**	0.2563 (0.0785)**	0.0419 (0.0193)	0.0093;0.0860	58.19%
	Men	133	-0.1079 (0.1213)	-0.2084 (0.1194)	0.2445 (0.0899)**	0.4114 (0.1154)***	0.1006 (0.0469)	0.0187;0,2030	-93.23%
	Women	232	0.1935 (0.1112)	0.1724 (0.1114)	0.1188 (0.0710)	0.1772 (0.1043)	0.0211 (0.0176)	-0.0065;0.0616	No mediation

Observations: The variables in the mediation model were HbA1c (X), SAF (M) and a-PWV (Y). The covariables selected in the adjusted model were age, sex, smoking status, insulin level, SBP, BMI and educational level. The subsample was derived from the full sample by excluding participants identified as having definite CV risk according to SAF risk. Statistical significance is indicated by asterisks, where * indicates $p \leq 0.05$, ** indicates $p \leq 0.01$ and *** indicates $p \leq 0.001$.

Abbreviations: CI, confidence interval; IE, indirect effect; M: mediator variable; n, sample size; SE, standard error; X, independent variable; Y, dependent variable.

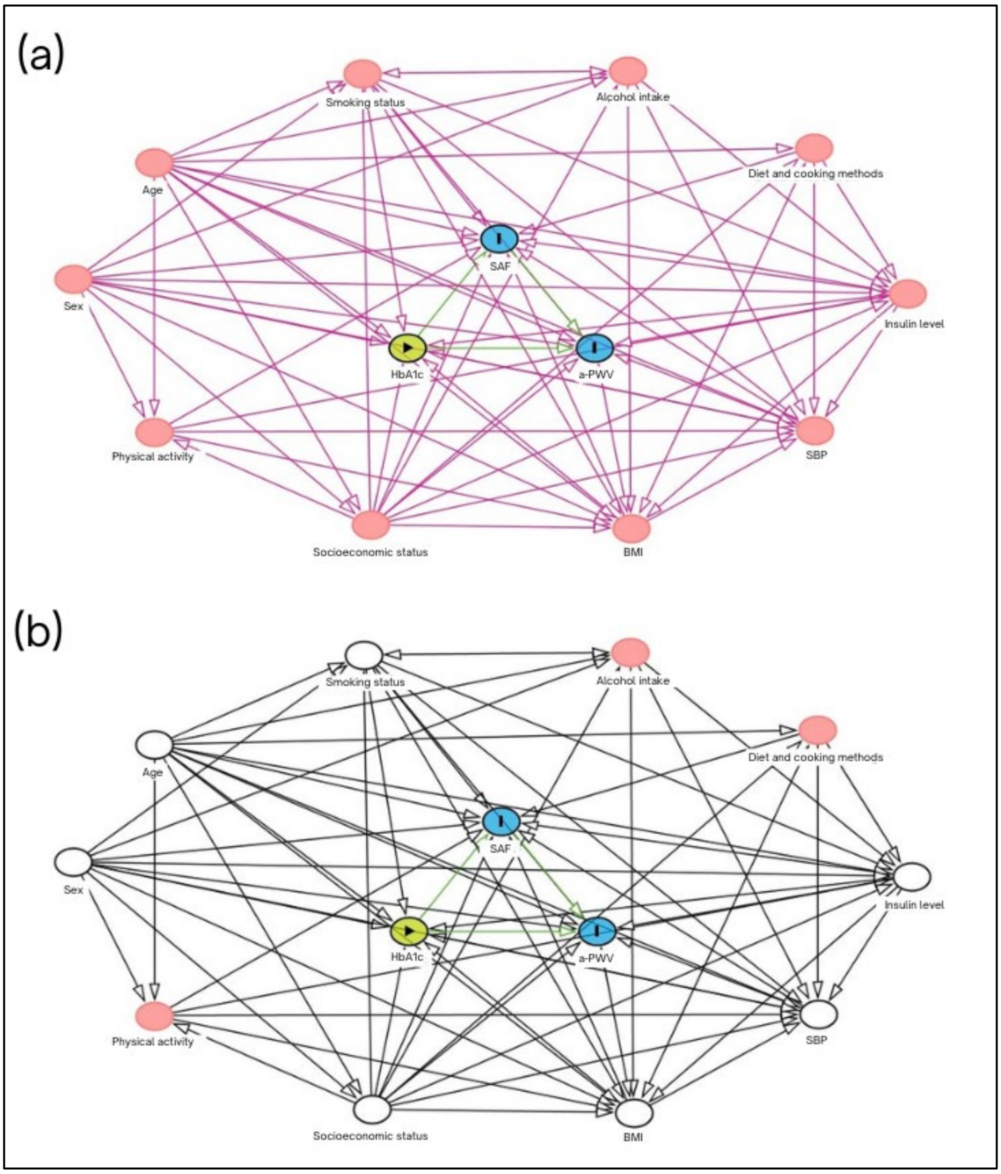

Figure S1. Directed acyclic graphs (DAGs) on the causal structure of the relationship between HbA1c (exposure, green) and a-PWV (outcome, blue) and its possible mediating variable, SAF (mediator, blue).

(a) Figure "a" shows the directed acyclic graph (DAG) for the causal structure of the relationship between HbA1c (exposure, green) and PWV (outcome, blue) and its possible mediating variable SAF (mediator, blue). The ancestral variables of the exposure, outcome and mediator variables are represented by pink circles. The causal pathways are represented by green arrows, and the polarisation pathways are represented by pink arrows.

(b) Figure "b" depicts the main DAG after adjusting by the minimum sufficient adjustment set for the total effect. Variables that are part of the minimum sufficient adjustment set are indicated in white (age, sex, smoking status, insulin level, systolic blood pressure, body mass index and socioeconomic status), whereas variables that do not need to be adjusted are indicated in pink (physical activity, alcohol intake, diet and cooking methods). The bias pathways have been eliminated (pink arrows have been replaced with black arrows, indicating effective control of relevant confounders), and only the 'causal' pathways remain open (both direct and indirect pathways, i.e., via mediators).

Observations: As outlined in the Methods section, owing to the unavailability of data on socioeconomic status, we opted to utilise a closely related variable, educational level, which has been demonstrated to have a strong correlation with socioeconomic status. This approach ensures the reliability of the analysis.

Abbreviations: a-PWV, aortic pulse wave velocity; BMI, body mass index; DAG, directed acyclic graph; HbA1c, glycated haemoglobin; SAF, skin autofluorescence; SBP, systolic blood pressure.

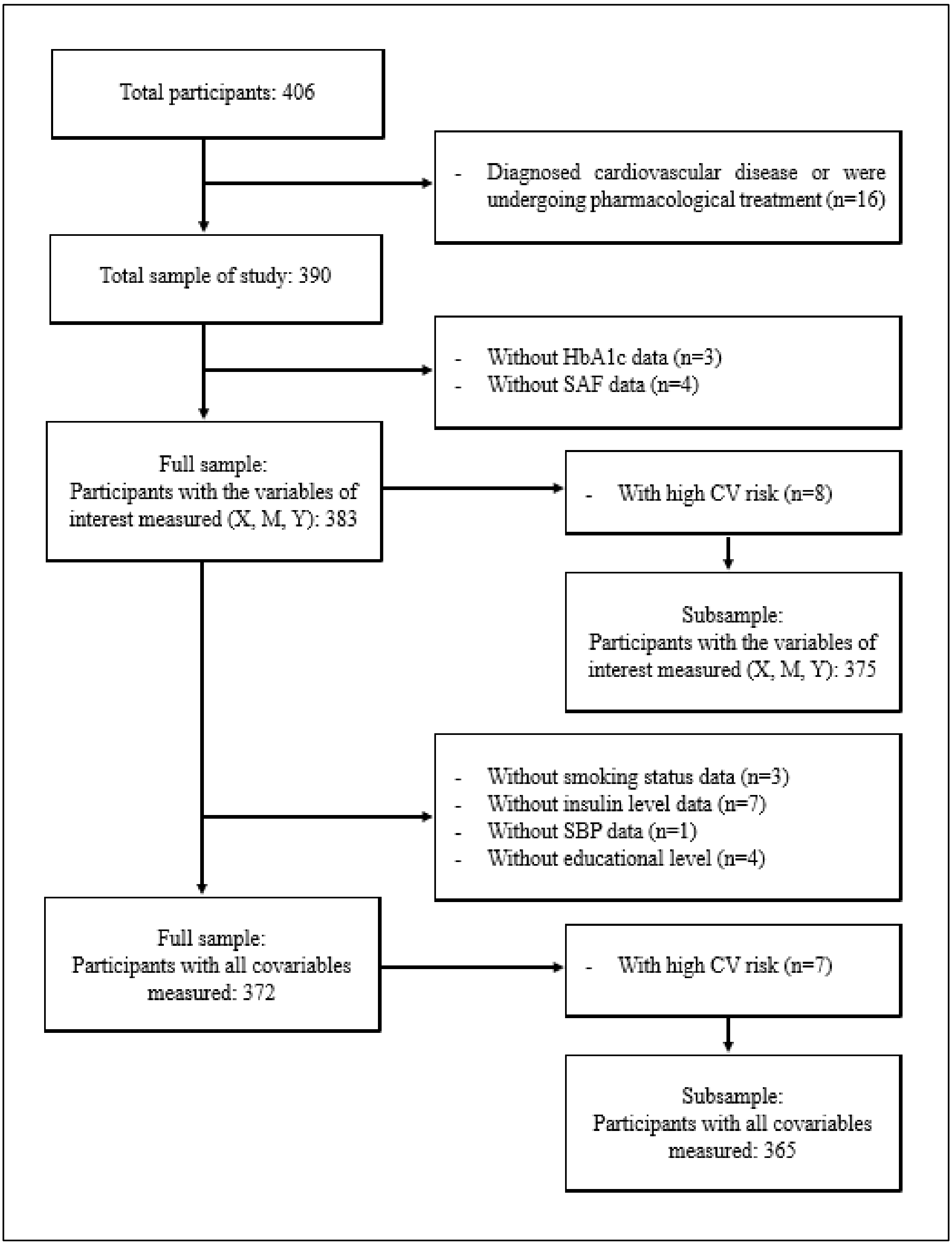

Figure S2. Flow chart of the study participants in the current study from the original EVasCu study.

Observations: The covariables selected were age, sex, smoking status, insulin level, SBP, BMI and educational level. The variables of interest were HbA1c (X), SAF (M) and a-PWV (Y).

Abbreviations: a-PWV, aortic pulse wave velocity; CV, cardiovascular; BMI, body mass index; HbA1c, glycated haemoglobin; M: Mediator variable; SAF, skin autofluorescence; SBP, systolic blood pressure; X, independent variable; Y, dependent variable.
